# A Concise Review of the Conflicting Roles of Dopamine-1 versus Dopamine-2 Receptors in Wound Healing

**DOI:** 10.3390/molecules23010050

**Published:** 2017-12-26

**Authors:** Alexandra R. Vaughn, Michael James Davis, Raja K. Sivamani, Roslyn Rivkah Isseroff

**Affiliations:** 1 Department of Dermatology, University of California–Davis, Sacramento, CA 95816, USA; arv42@drexel.edu (A.R.V.); rksivamani@ucdavis.edu (R.K.S.); 2 Drexel University College of Medicine, Philadelphia, PA 19129, USA; 3 Emory University School of Medicine, Atlanta, GA 30307, USA; michael.james.davis@emory.edu; 4 Department of Biological Sciences, California State University–Sacramento, Sacramento, CA 95819, USA; 5 Veterans Administration, Northern California Health Care System, Department of Dermatology, Mather, CA 95655, USA

**Keywords:** wounds, dopamine, wound healing

## Abstract

Catecholamines play an important regulatory role in cutaneous wound healing. The exact role of dopamine in human epidermis has yet to be fully elucidated. Current published evidence describes its differential effects on two separate families of G protein coupled receptors: D1-like and D2-like dopamine receptors. Dopamine may enhance angiogenesis and wound healing through its action on dopamine D1 receptors, while impairing wound healing when activating D2 receptors. This review summarizes the evidence for the role of dopamine in wound healing and describes potential mechanisms behind its action on D1 versus D2-like receptors in the skin.

## 1. Introduction

Wound healing occurs as a normal physiological response to cutaneous damage, either from acute trauma or underlying disease, leading to skin breakdown and ulceration. The process of cutaneous wound healing consists of several phases, including an inflammatory phase, a proliferative phase, and a remodeling phase, all of which rely on communication of various cells and signaling molecules. Any defect in the steps involved can impair tissue regeneration and lead to poor wound healing.

The skin contains a rich supply of nerves, with nerve endings in the dermis, extending into the epidermis, as well as surrounding blood vessels, pilosebaceous units, and sweat glands. Several studies have shown that nerves play an important regulatory role in cutaneous wound healing [1,2,3]. Nerves can contribute to the inflammatory response in the first phase of wound healing, by releasing endogenous catecholamine neurotransmitters, such as epinephrine, norepinephrine, and dopamine (DA) [4]. Human keratinocytes also have the capability to utilize L-tyrosine to synthesize L-DOPA, subsequently resulting in the production of dopamine, norepinephrine, and epinephrine [4]. DA is not only pivotally important in the central nervous system for emotion, cognition, and movement, but it also has peripheral effects in regulating endocrine, cardiovascular, renal, gastrointestinal, and immune system functions [5,6].

**Figure 1:**
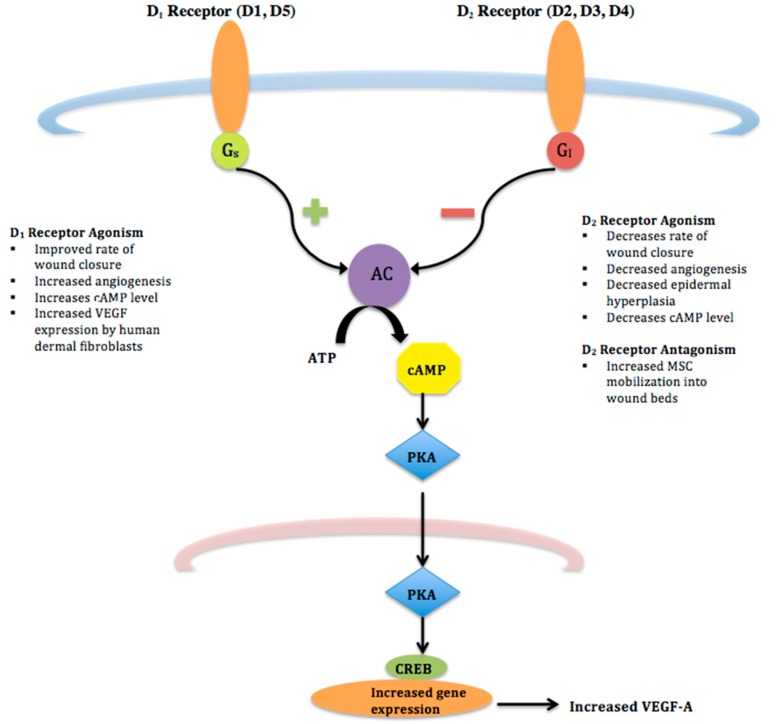
D_1_ and D_2_ Receptor Pathways–D_1_ and D_2_ receptors are G-protein coupled receptors that signal through different cascades. D_1_ receptor agonists lead to increased production of adenylyl cyclase (AC) via a G_s_ pathway. Conversely, agonism of D_2_ receptors stimulates G_i_ proteins leading to decreased cAMP and inhibiting neuronal activity.

DA effects are mediated by five DA receptor subtypes, each a G-protein coupled receptor, with two distinct signaling mechanisms that divides them into two separate families. Agonists of D1-like receptors (D1 and D5 receptors) activate G_s_ proteins, which activate adenylyl cyclase and increase the intracellular concentration of cAMP. On the other hand, D2-like receptor (D2, D3, D4 receptors) agonists stimulate G_0_/G_i_ proteins, thereby down regulating the intracellular cAMP pathway and inhibiting neuronal activity (See Figure 1) [7]. Hereafter, D1-like and D2-like receptors will be referred to as D_1_ and D_2_ receptors, respectively.

DA receptors are also expressed on various cell types within the skin [8,9,10]. Fuziwara, et al. demonstrated that DA plays a role in murine skin barrier homeostasis by acting on D_2_ receptors that are present on epidermal keratinocytes, resulting in changes in their cAMP level that regulate barrier function [11]. This study found differential expression of the D2 and D4 receptors within the basal and outermost layers of the epidermis [11]. Other work has demonstrated the presence of D_1_ receptors in dermal fibroblasts [9], and D_2_ receptors in endothelial cells [12]. Dopamine reaches the skin primarily via nerve endings, but there is also evidence to suggest that keratinocytes express enzymes to both synthesize and metabolize dopamine [11,13]. DA receptors have been identified on B cell and natural killer cell membranes, and to a lesser extent, on T cells and monocytes [14]. However, the literature on these cells is vast and conflicting, and is therefore not included in this review.

DA’s role in cutaneous wound healing has become an important focus of investigation, although it is hard to ascribe to it a strictly pro- or anti-reparative role because of divergent reported effects. The purpose of this review is to highlight and summarize the evidence of dopamine’s role in wound healing and to discuss potential mechanisms that underpin dopamine’s contribution to healing of wounds.

## 2. Influence on Angiogenesis and Dermal Wound Healing

One necessary component of successful wound healing is angiogenesis, the formation of new blood vessels. Angiogenesis occurs not only in wounds, but also during tissue remodeling in healthy and diseased states. A delicate balance exists between pro-angiogenic and anti-angiogenic factors, which dictates the formation of new blood vessels [15]. Angiogenesis is vital to the regeneration of new tissue in wound beds by providing adequate nutrients and oxygen to aide formation of granulation tissue [16,17]. It is mediated by VEGF-A, the primary cytokine stimulating the growth of neovessels in wounds, in which it acts in a paracrine manner on VEGF-2 receptors in endothelial progenitor cells and endothelial cells [18,19]. Both pro- and anti angiogenic responses and changes in VEGF-A in response to activation of the DA receptors have been reported, and the differences are likely related to the DA receptor being targeted and the cells in which they have been investigated.

Using eticlopride, a specific, a specific D_2_ receptor antagonist, Shome, et al. demonstrated that excisional wounds in mice treated with this drug had a dose-dependent increase in wound closure (maximally 78.3% by day 7, as compared to 38.2% in control mice) that was associated with a statistically significant increase in wound microvessel density [20]. To further probe these results, these investigators examined the expression of HoxD3, a transcription factor that is involved in angiogenesis [21,22], and one of the genes for which it modulates expression, α5β1 integrin [23]. They found that eticlopride treatment increased HoxD3 expression and up-regulated α5β1 integrin in wound bed tissue. Examining cultured human umbilical vascular endothelial cells (HUVECs) directly, they found that treatment with 1 μM of DA (physiological concentrations at the neuronal synapse) [24] blocked the vascular endothelial growth factor A (VEGF-A) -induced expression of HoxD3 and α5β1 integrin expression, while conversely, treatment with eticlopride restored HoxD3 and α5β1 integrin expression. Since these studies were performed in HUVECs, rather than in murine dermal angiogenic endothelial cells that would have been more relevant to the in vivo murine model, translating the in vitro results to the in vivo findings may be challenging. Dopamine antagonists are widely used clinically as antipsychotic drugs, so one could envision analysis of incidence of chronic wounds in this patient population to determine if there is any association between the DA receptor antagonist use and improved healing.

As opposed to receptor antagonism, the role of direct activation of the D_1_ receptor in wound healing and angiogenesis was evaluated by Chakroborty et al. [9]. Excisional wounds in streptozotocin-induced (type 1 model) diabetic mice healed more quickly in animals treated for five days with specific D_1_ receptor agonists (SKF38393 or SKF81297), as compared to controls. By day 11, wound closure reached 100% in the D_1_ receptor agonist treated mice, while wound closure only reached 42% in the non-treated control mice. Furthermore, D_1_ receptor activation significantly increased the expression of VEGF-A and angiogenesis in wounds of the diabetic mice when compared to the untreated mice. Similar results were seen in the db/db (type 2 model) diabetic mice. Interestingly, they found that the source of the increased VEGF-A in the wound was the dermal fibroblast. Human dermal fibroblasts, isolated from the skin of diabetic patients increased their VEGF-A production in response to D_1_ agonists. This was a surprising finding, since prior work had shown that D_2_ activation of endothelial cells decreases, rather than increases, VEGF-A generation [25,26]. Thus, not only are there opposing responses to activation of different receptor types, but also different cell types expressing the same receptor type can respond with directly opposing responses.

## 3. Influence on Epidermis and Keratinocytes

Outside of the work demonstrating the role of DA in the epithelial pigmentation, there are remarkably few studies investigating other potential roles in the epithelium. This is particularly surprising given that epidermal keratinocytes express DA receptors [11], as well as synthesize DA [13]. One of the earliest studies in this area was that of Harper and Flaxman [27], who noted that dopamine decreased keratinocyte mitosis in human epidermis and ascribed this to modulation of cAMP levels. The more recent work of Fuziwara, et al. demonstrated that activation of the D_2_ receptor with topical application of its agonist, bromocriptine, decreased epidermal proliferation, while the D_2_ receptor antagonist, L-741626, conversely induced epidermal hyperplasia in barrier-disrupted mouse skin, with an associated decrease in cAMP with bromocriptine treatment, and increased cAMP with the L-741626 treatment [11]. This is in agreement with other studies that have noted that increased cAMP levels associated with decreased keratinocyte mitosis and proliferation [28,29,30].

On the other hand, this group found that the D_2_ agonist significantly accelerated barrier recovery after tape stripping, while D_2_ receptor antagonists (L-741626, L-741742, and remoxidride) significantly slowed the rate of barrier recovery. Since these changes were associated with decreased (agonist treatment) or increased (antagonist treatment) cAMP levels, these findings extended the group’s earlier work that noted that increased cAMP levels are associated with skin barrier disruption, and the restoration of the barrier is associated with cAMP lowering drugs [31,32,33]. In addition, the researchers further hypothesized that since D_2_ receptor antagonists delayed barrier repair, there must be endogenously produced dopamine by keratinocytes, which was confirmed based on an increase in DA level in the culture medium after keratinocyte incubation [11], confirming earlier findings of DA generation by keratinocytes [13,34]. It is tempting to hypothesize that dopamine is secreted by keratinocytes in response to barrier disruption or injury, and by virtue of its effects on the D_2_ receptor localized in the uppermost layer of the epidermis, can hasten barrier repair and the protective function of the epidermis, even at the cost of decreasing basal keratinocyte proliferation. However, further experiments are needed to fully understand the dopamine-receptor interaction in human epidermis.

Additionally, DA can alter the innate immune responses of keratinocytes by stimulating their production of Il-6 and Il-8, which could also be a factor in modulation of the inflammatory response required in the early phases of wound healing [35].

## 4. Influence on Mesenchymal Stem Cells

Wound healing requires the interaction of various regulatory molecules that are generated by multiple cell types, such as endothelial cells, endothelial progenitor cells, and mesenchymal stem cells (MSCs) [15,17,36,37]. MSCs, originating from a number of tissues including adipose tissue and adult bone marrow, mobilize in response to chemokines that are released from various distant sites in the body. In the case of wounds, the release of wound-generated growth factors and cytokines mobilize and attract MSCs to the injured tissue [38,39]. This is important in cutaneous wound healing, because MSCs can induce angiogenesis in healing tissues [37,40,41]. Therefore, it is important to understand how dopamine can influence MSC biology.

DA D_2_ receptor antagonism enhances mobilization of MSCs into wound tissue beds [42]. Shome et al. compared the numbers of MSCs (CD34^−^, CD35^−^, CD105^+^) in peripheral blood in D_2_ antagonist treated mice versus controls [42]. Wounded mice that were treated with eticlopride (a specific D_2_ receptor antagonist) had significantly higher numbers of peripheral MSCs than control mice. Shone et al. confirmed that approximately 86% of the MSC population did indeed express DA D_2_ receptors. By tracking the migration of BrdU-labeled bone marrow-derived MSCs injected into the systemic circulation, they demonstrated that a significantly higher number of BrdU-labeled MSCs trafficked to the wound beds of antagonist (eticlopride) treated mice as compared to control mice [42].

VEGF-A is a key growth factor involved in regulating angiogenesis within wound tissue [43,44], and chemotaxis of human MSCs [45]. Evidence supports that VEGF-A deficiency and poor angiogenesis are important pathogenic mechanisms that underpin the delayed wound healing in people with diabetes mellitus [46,47]. When 1 μM of DA was added to murine MSCs, VEGF-A induced MSC migration was significantly inhibited. However, when the MSCs were pre-treated with 100 μM of eticlopride, DA’s inhibitory effect was reversed [42]. The investigators found that DA prevents VEGF-A induced MSC migration by suppressing the phosphorylation of VEGFR-2 receptors and phosphorylation of Akt. It was not clear why the authors had not reported an untreated control in evaluating phosphorylation of VEGFR-2 and Akt as this would have allowed for a better assessment of the influence of DA independent of VEGF-A stimulation. Regardless, DA appears to inhibit VEGFR-2 and Akt phorphorylation in VEGF stimulated MSCs, which is reversed by blockade of the D_2_ dopamine receptor.

## 5. Summary

The significance of catecholamines in epidermal function was postulated over 60 years ago, but the exact role of dopamine has yet to be fully elucidated. Disparate actions of DA on D_1_ versus D_2_ receptors in human skin have led to the question of the predominant role of DA in wound healing (See Table 1).

Regarding angiogenesis, the D_1_ and D_2_ receptors appear to respond differently: angiogenesis is improved by activation of the D_1_ receptor by its agonists, while for the D_2_ receptor, it is antagonism that improves angiogenesis. D_1_ receptor agonists appear to improve angiogenesis by inducing VEGF synthesis by fibroblasts. D_2_ receptors appear to have differential effects in the dermis and the epidermis as well. In the epidermis, D_2_ receptor agonists improve skin barrier repair and reduce proliferation of the keratinocytes. On the other hand, D_2_ receptor antagonists improve dermal angiogenesis, wound healing, and MSC trafficking to the wound bed. It is clear that fully characterizing the DA pathway and D_1_ receptor in the skin is needed to provide therapeutic insight. A thorough understanding of catecholamine behavior in the wound microenvironment is important in the setting of wound healing, and the development of new therapeutic agents to specifically target D_1_ receptors could be investigated for use in wound healing. Since both DA agonists and antagonists are approved as drugs for clinical use for other indications, the pathway to translating these drugs to wound healing indications could be relatively facilitated. We look forward to those developments.

## Figures and Tables

**Table 1:** Differential Effects on Wound Healing Mediated by DA D_1_ versus D_2_ Receptors.

	Agonism of D_1_ Receptors	Agonism of D_2_ Receptors	Antagonism of D_2_ Receptors
Wound Closure	Increases [9]	Decreases	Increases [20]
Angiogenesis	Increases [9]	Decreases [11]	Increases [20]
Epidermal hyperplasia	-	Decreases [11]	
MSC Mobilization into wound beds			Increases [11]
VEGF-A Expression	Increases [9]	Decreases [25,26]	
Cyclic AMP level	Increases [7]	Decreases [11]	
Keratinocyte mitosis	Exogenous DA inhibits keratinocyte mitosis in vitro [27]		
